# OCT-based deep-learning models for the identification of retinal key signs

**DOI:** 10.1038/s41598-023-41362-4

**Published:** 2023-09-05

**Authors:** Inferrera Leandro, Borsatti Lorenzo, Miladinovic Aleksandar, Marangoni Dario, Giglio Rosa, Accardo Agostino, Tognetto Daniele

**Affiliations:** 1 Department of Medicine, Surgery and Health Sciences, Eye Clinic, Ophthalmology Clinic, University of Trieste, Piazza Dell’Ospitale 1, 34125 Trieste, Italy; 2 Institute for Maternal and Child Health IRCCS “Burlo Garofolo”, Trieste, Italy; 3 Department of Engineering and Architecture, University of Trieste, Trieste, Italy

**Keywords:** Diseases, Health care, Engineering, Biomedical engineering, Signs and symptoms, Eye manifestations, Biomarkers, Diagnostic markers, Predictive markers, Prognostic markers, Medical research, Biomarkers, Translational research

## Abstract

A new system based on binary Deep Learning (DL) convolutional neural networks has been developed to recognize specific retinal abnormality signs on Optical Coherence Tomography (OCT) images useful for clinical practice. Images from the local hospital database were retrospectively selected from 2017 to 2022. Images were labeled by two retinal specialists and included central fovea cross-section OCTs. Nine models were developed using the Visual Geometry Group 16 architecture to distinguish healthy versus abnormal retinas and to identify eight different retinal abnormality signs. A total of 21,500 OCT images were screened, and 10,770 central fovea cross-section OCTs were included in the study. The system achieved high accuracy in identifying healthy retinas and specific pathological signs, ranging from 93 to 99%. Accurately detecting abnormal retinal signs from OCT images is crucial for patient care. This study aimed to identify specific signs related to retinal pathologies, aiding ophthalmologists in diagnosis. The high-accuracy system identified healthy retinas and pathological signs, making it a useful diagnostic aid. Labelled OCT images remain a challenge, but our approach reduces dataset creation time and shows DL models’ potential to improve ocular pathology diagnosis and clinical decision-making.

## Introduction

A large part of clinical data consists of medical images that might contain relevant features that are not visible to the human eye. Thus, there is a growing interest in the development of computer-aided systems for the automated examination of OCT images useful to support ophthalmologists in diagnosis. OCT images are fundamental for the diagnosis of numerous retinal diseases, being able to provide detailed information about all retinal layers.

Deep learning (DL), a method of Machine Learning (ML), is changing the approach to the diagnosis and management of different medical pathologies since advanced DL techniques can detect pathological characteristics^1,2^. In particular, ML algorithms are powerful tools in the automatic detection and quantification of retinal biomarkers identified on OCT^3–5^. In the last years, different ML models were developed and widely used for the recognition of OCT images acquired on patients with major eye pathologies such as diabetic retinopathy (DR), age-related macular degeneration (AMD), central serous chorioretinopathy (CSC), epiretinal membrane (ERM) and glaucoma^6–16^.

Regarding OCT images classification, the most used CNN architectures are VGG, ResNet and Inception, and have shown very promising results so far^17–21^.

Despite the promising results given by the literature on the use of the VGG-16, ResNet-50, and Inception-v3 architectures for the classification of OCT images, the need for large data sets and non-standardized image acquisition techniques limits the applicability of ML in the clinical domain^22^. Furthermore, a low diffusion of ML-based decision-making in healthcare should be underlined, mainly due to a lack of interpretability of the classification process related to DL-based methods^23^. In fact, decision-making by VGG-16 as well as by other DL architectures happens in a black-box mode, i.e. without having evidence of the process that led to a certain result.

To overcome some of the challenges of clinical applicability/interpretability and the requirement of large and balanced datasets of the DL, our study focused not on direct pathology classification, but on the identification of retinal abnormality signs by using the DL approach applied to OCT images.

The detection of abnormalities provides direct insight into the presence of one or more signs that can be used by ophthalmologists as a guide in the decision process. Thus, the automatic identification of retinal abnormality signs from OCT images is a fundamental building block in developing a first step of an interpretable decision support system for the diagnosis of retinal pathologies.

Our study aimed to identify the presence of one or more of the following abnormality signs: epiretinal membrane (ERM), intraretinal fluid (IF), subretinal fluid (SF), drusen (D), macular neovascularization (MNV), vitreomacular adhesion (VMA), macular hole (MH) and backscattering (BS).

The identification of singular abnormality signs makes it possible to imitate the deductive process used by the ophthalmologist to diagnose ocular pathologies rather than relying exclusively on the outcome (pathological or not) of a black-box model as that based on DL. This approach also reduces the overall number of images generally necessary to identify different pathologies.

## Materials and methods

This retrospective observational study was conducted at the University Eye Clinic of Trieste. All patients enrolled in the study signed an informed consent to use the data. The retrospective study was carried out following the principles of the Declaration of Helsinki, and the research protocol received approval from the Regional Ethics Committee (CEUR) of Friuli Venezia Giulia, Italy (protocol n. 17,094/2022).

### Data collection

Completely anonymized OCT scans in A line-scans protocol of 9.0 mm length were retrospectively analyzed. Images were acquired by Spectralis OCT (Heidelberg Engineering, Heidelberg, Germany) with 815 nm laser source, 3.9 μm/pixel axial resolution, 5.7 μm/pixel lateral resolution and 768 × 496 pixel image size.

The study included horizontal and vertical line scans, centered on the fovea, of healthy and pathological eyes, of adults between 18 and 95 years old, acquired from January 2017 to September 2022.

The inclusion criteria for the pathological group were the presence of one or more of the following signs: ERM, IF, SF, D, MNV, VMA, MH and BS. The healthy group consisted of individuals who did not present any retinal abnormal sign on OCT scans. Poor quality images (Spectralis Quality parameter lower than 23) were excluded.

### Image labeling and preprocessing

All images were examined and labelled by two experienced retinal specialists (LI,DM). Poor quality images, OCT scans outside the foveal area and images for which an agreement was not reached between the two specialists were excluded from the dataset.

**Figure 1:**
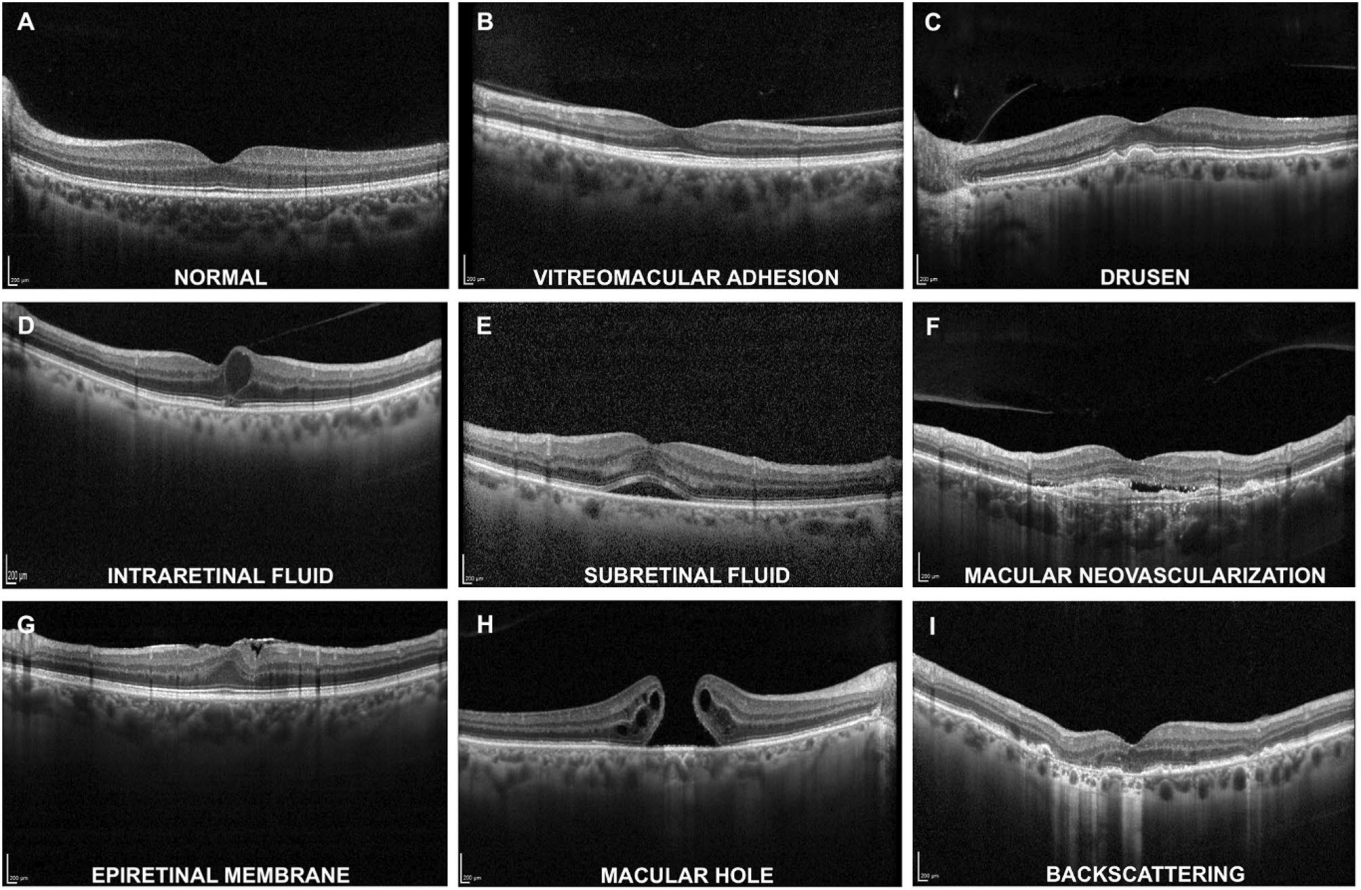
Representative OCT images of retinal signs included in the study.

Representative OCT images of each sign are shown in Fig. 1. Each image was cropped in the central area of the scan to 621*445 pixels and then resized to 224*224 pixels, to obtain the default input image size for the VGG-16 convolutional neural networks algorithm. The resizing was accomplished by using a bicubic interpolation.

### Datasets population and training process

The labeled images were preprocessed to create 9 predictive binary models. The first model was trained to identify scans belonging to the healthy or pathological group, while the remaining 8 models were used to further identify each of the signs of retinal degeneration. In the first model, the group of all images belonging to healthy eyes and the group of images containing at least one sign was used. In the other eight cases, the group of images including one specific sign and the group containing images lacking that sign was considered. To have a balanced dataset, for each model, the number of images considered was the same for each group. The 10% of images coming from healthy as well as the 10% of images of each sign were randomly selected and used as the test set. The remaining 90% of images was used for the fivefold cross-validation. Table 1 reports the number of images containing one or more abnormal signs.

**Table 1:** Number of images containing one or more abnormal signs.

# of signs	BS	MNV	D	IF	SF	MH	ERM	VMA	Total images
1	212	96	1091	964	265	145	880	1733	5386
2	418	375	470	727	302	250	518	342	1701
3	257	245	95	296	130	80	156	70	443
4	83	88	37	88	52	12	27	33	105
5	15	15	5	11	13	2	6	8	15
Total	985	819	1698	2086	762	489	1587	2186	7650

### Modeling

Among the three most used CNN architectures, in this study, we selected VGG-16 because it presents a low number of hidden layers and a small convolution filter (3 × 3), thus requiring a small training data set, probably reducing the network’s tendency to overfit during training.

The selection of VGG has been proven effective in imaging for medical diagnosis. The review of analyzed the trends in the application of deep learning networks in medical image analysis between 2012 and 2020, and found that VGG was among the three most frequently used Convolutional neural network-derived networks applied in medical image analysis^24^. The architecture was used to diagnose Choroidal Neovascularization (CNV) in retinal OCT images with an accuracy of approximately 97.5^25^. In the classification of diabetic retinopathy, the modified VGG16 has been proposed and outperformed state-of-the-art methods in terms of accuracy and computational resource utilization^26^. Outside of ophthalmology, VGG16 has also been applied in the classification of breast cancer using mammography images, achieving a test score of 88%^27^. Additionally, VGG16 has been utilized in brain tumor detection through MRI, achieving a high accuracy of about 96.1% UNet-VGG16 with transfer learning for MRI-based brain tumor segmentation^28^. In the field of breast histopathology image analysis, VGG16 has been used as a pre-trained model to extract high-level features for breast cancer classification^29^. Furthermore, a modified version of VGG16, has been proposed for the classification of pneumonia X-ray images, demonstrating superior outcomes compared to other convolutional neural networks^30^.

**Figure 2:**
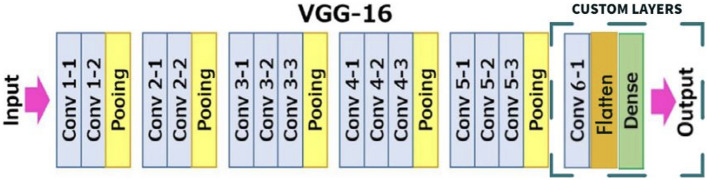
Modified VGG-16 model used for each classifier.

As the goal was to obtain nine binary classifiers, we used the modified VGG-16 model depicted in Fig. 2.

Each of the nine binary models was developed using transfer learning and fine-tuning techniques on the pre-trained model (VGG-16). To build the model, the top (rightmost in Fig. 2) layers of the VGG-16 were replaced by a custom layer, and the sigmoid dense layer was used for classification, while the previous layers were kept frozen. However, since dense layers take 1D vectors as input, while the output of previous layers is 3D tensors, a flattened layer converting data into a 1-dimensional array was used.

The new layers can learn patterns from previously learnt convolutional layers because a very small learning rate is utilized (Adaptive Moment Estimation Algorithm (ADAM) with a learning rate of 0.0001)^31^.

By applying this approach, the retinal abnormality signs could be recognized even if the pre-trained VGG-16 were not trained using our images. For training each model, the images were resized and augmented using typical data augmentation techniques. Once this was done, we flowed them in batches of 32 into the model and started the training. Each model was trained through two steps: in the first step, the model was trained with frozen convolution layers for adjusting the top layers (transfer learning). In the second step, the early stopping technique was used if after eight epochs there is no improvement in the accuracy measured on the validation set.

Each model was trained for a maximum of 70 epochs: 40 epochs for the transfer learning phase and 30 epochs for fine-tuning, always using batches of 32 elements. The number of maximum epochs was determined empirically in preliminary trials after recording the number of steps the model needed to converge. The early Stopping technique was applied to monitor the accuracy of the models for each epoch on the validation datasets and to terminate the process when the performances did not further improve. At the end of the training, the model with the best performance on the validation set was selected and tested on the test sets.

Models were trained using Python version 3.10 and Keras, a high-level API of Tensorflow 2, on a computer equipped with Ryzen 7 2700 processor, NVIDIA RTX 3070ti graphic card and 16 GB DDR4 ram.

### Evaluation metrics

Confusion matrices were generated to understand the detail of the misinterpretations and to evaluate the performance of the model by computing the following metrics: accuracy, sensitivity and specificity, and area under the ROC curve (AUC). Cohen's Kappa indexes were obtained to examine the agreement between the systems with the ground truth on the assignment of categories of labelled variables. All analyses were carried out through the Python library scikit-learn^32^.

### Model visualization (GRAD-CAM)

**Figure 3:**
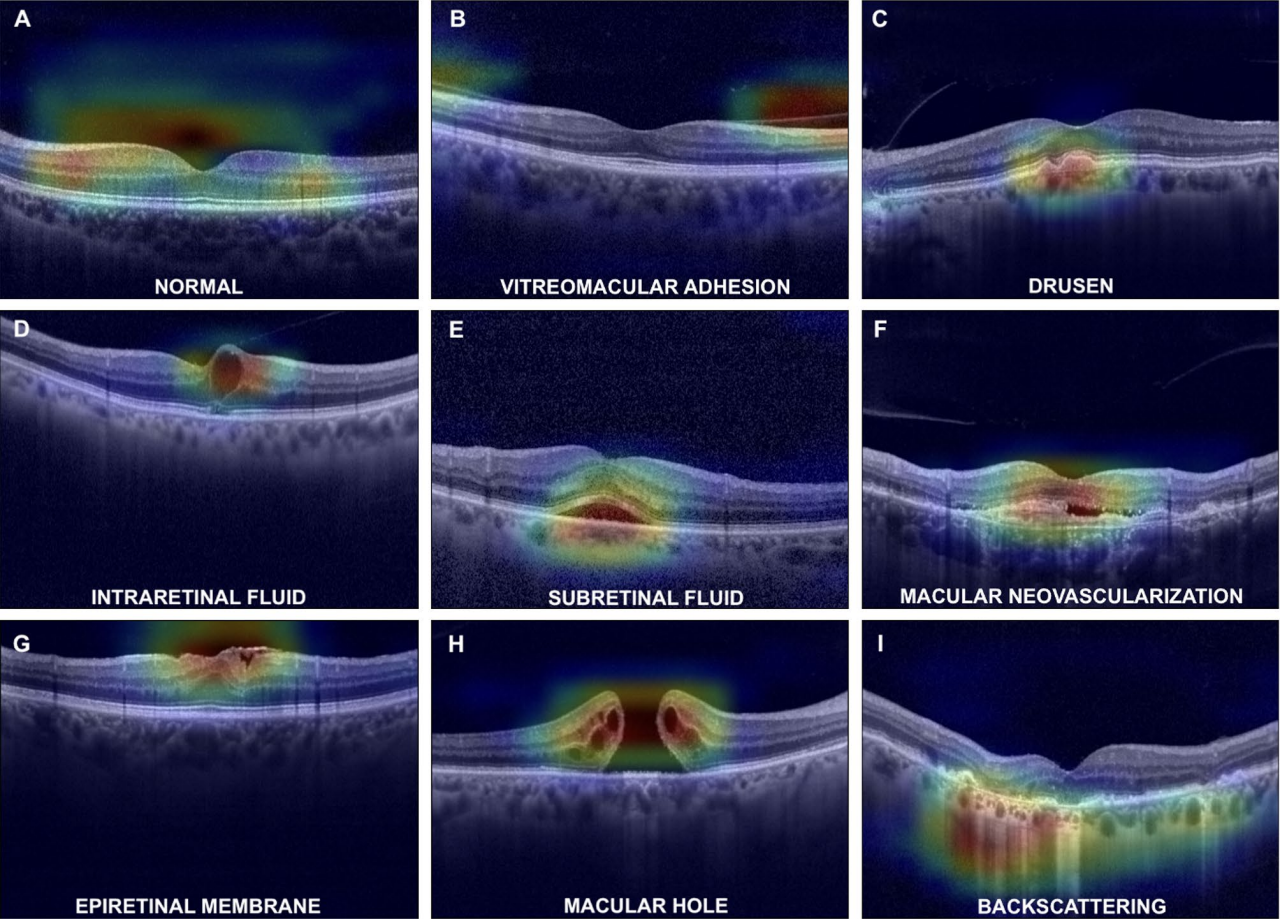
Grad-CAM images for each retinal finding.

To understand the CNN predictions, Gradient-weighted class activation mapping (Grad-CAM heatmap) for each CNN model was used. Grad-CAMs were implemented before the last fully connected layer of VGG16 and allowed to highlight the regions most involved in the decision made by the model. The regions of interest or crucial features within the input data that influenced the model's decision were visually identified by generating heatmaps. Insights into the reasoning behind the model's predictions were gained with the help of this approach. Examples of Grad-CAM heat maps are shown in Fig. 3.

## Results

A total of 21,500 completely anonymized OCT scans of 11,245 patients (5258 Male and 5987 Female) with a mean age of 71.2 ± 16.5 were screened. These images were collected randomly from Heidelberg Spectralis OCT database. After this initial selection 10,770 images were included in the study. Of those, 3120 did not show any pathological sign and were marked as normal and 7650 were labelled as pathological, specifying the detected abnormality sign/s. Images presenting more than one sign were counted multiple times, thus 1587 ERM, 2086 IF, 762 SF, 1698 D, 819 MNV, 2186 VMA, 489 MH and 985 BS images, for a total of 10,612 images presenting one or more signs, were utilized.

**Figure 4:**
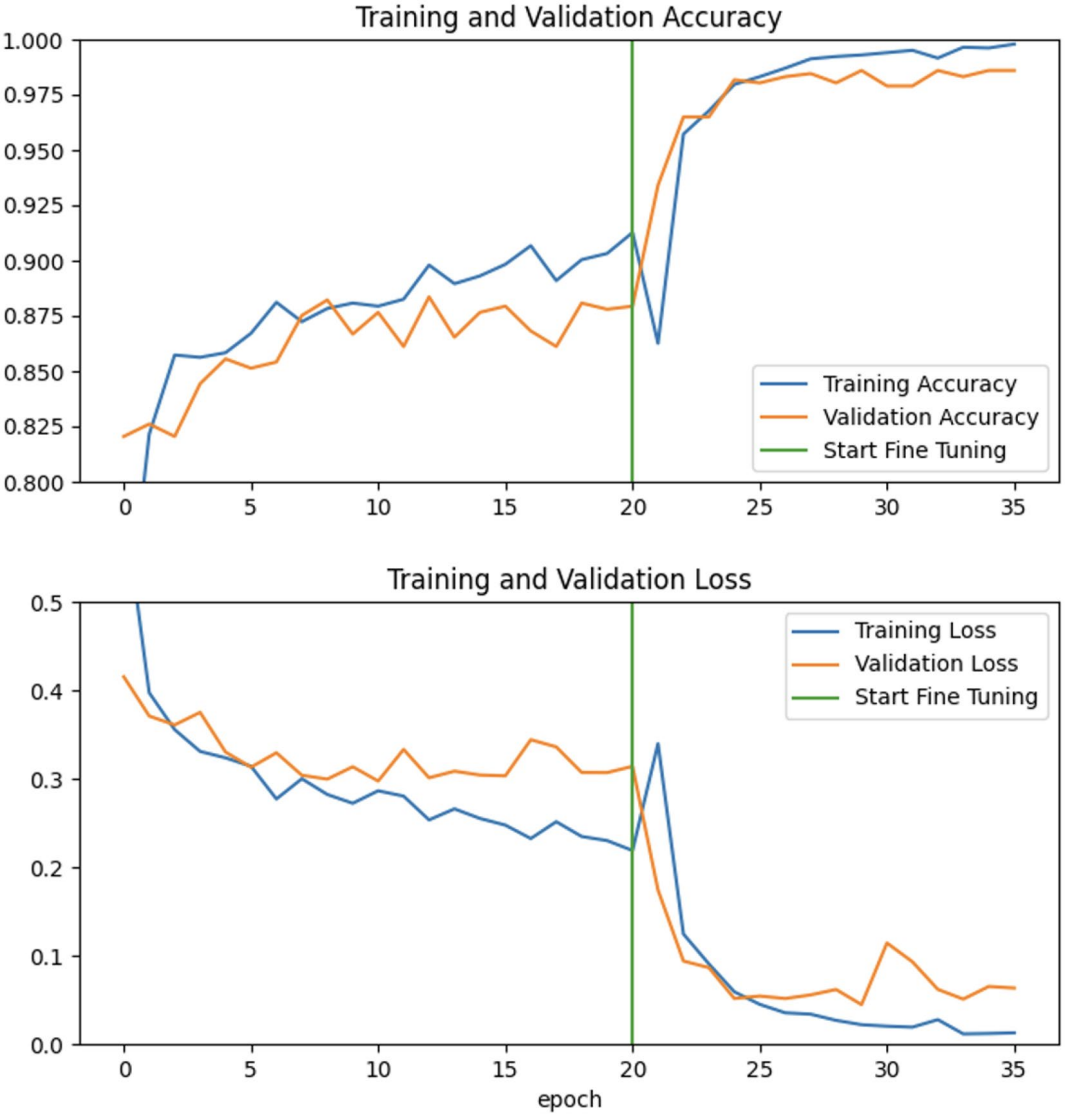
Example of a typical increase in the accuracy metric as well as the decrease of loss during the training phases.

Nine CNN models were created and trained to recognize an image as normal (no pathological signs) *vs.* pathological (presence of pathological signs), as well as to differentiate each pathological sign from the others. An example of a typical increase in the accuracy metric as well as the decrease in loss during the training phases is shown in Fig. 4.

Nine confusion matrices were calculated both on the validation and the test set. The results are reported in Tables 2 and 3. In each matrix, the rows represent the instances in the actual classes while the columns represent the instances in the predicted classes. Tables 4 and 5 show the accuracy, sensitivity, specificity, kappa value, and AUC for each of the nine CNN models calculated on the test and the validation set, respectively.

**Table 2:**
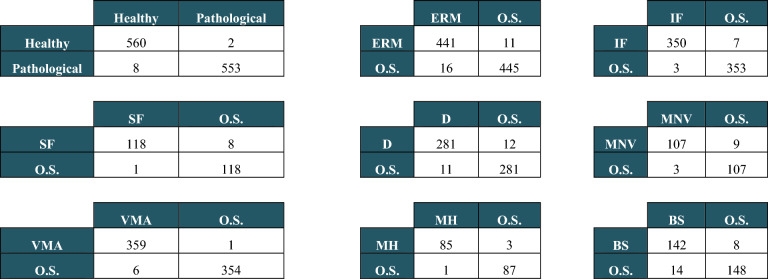
Confusion matrices obtained on the validation set for each model: Healthy vs Pathological, One sign (ERM, IF, SF, D, MNV, VMA, MH or BS) vs all Other Signs (O.S.).

**Table 3:**
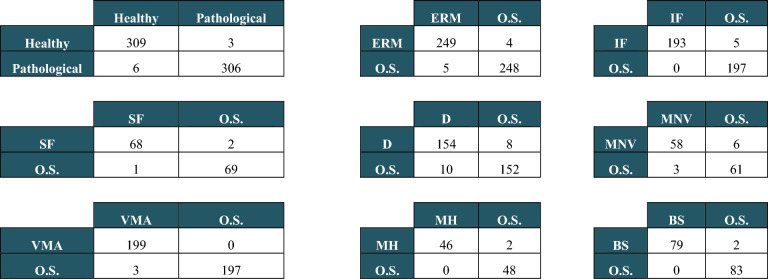
Confusion matrices obtained on the test set for each model: Healthy vs Pathological, One sign (ERM, IF, SF, D, MNV, VMA, MH or BS) vs all Other Signs (O.S.).

**Table 4:** Predictive values obtained from the nine models on the validation set.

	Accuracy	Sensitivity	Specificity	Kappa	AUC
Healthy	0.99	1.00	0.99	0.98	0.99
ERM	0.97	0.96	0.98	0.94	0.97
IF	0.99	0.98	0.99	0.97	0.99
SF	0.96	0.94	0.99	0.93	0.96
D	0.96	0.96	0.96	0.92	0.96
MNV	0.95	0.92	0.97	0.90	0.95
VMA	0.99	1.00	0.98	0.98	0.99
MH	0.98	0.96	0.99	0.95	0.98
BS	0.93	0.91	0.95	0.86	0.93

**Table 5:** Predictive values obtained from the nine models on the test set.

	Accuracy	Sensitivity	Specificity	Kappa	AUC
Healthy	0.99	0.99	0.98	0.97	0.99
ERM	0.98	0.98	0.98	0.96	0.98
IF	0.99	0.97	1.00	0.97	0.99
SF	0.98	0.97	0.99	0.96	0.98
D	0.94	0.95	0.94	0.89	0.94
MNV	0.93	0.91	0.95	0.86	0.93
VMA	0.99	1.00	0.98	0.98	0.99
MH	0.98	0.96	1.00	0.96	0.98
BS	0.94	0.92	0.96	0.88	0.94

**Figure 5:**
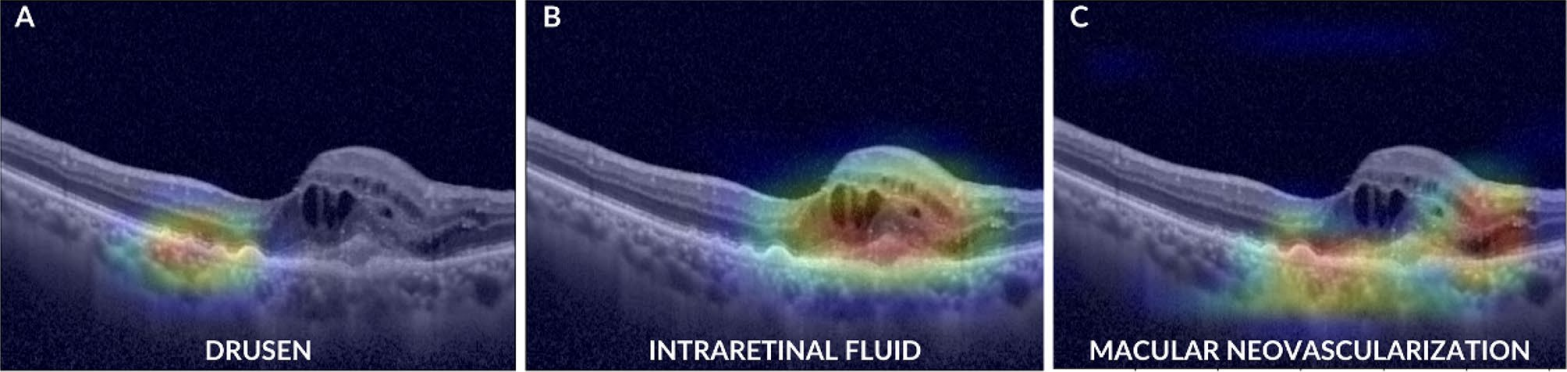
Grad-CAM images demonstrate the capacity of our CNNs to recognize multiple signs in the same OCT image.

**Figure 6:**
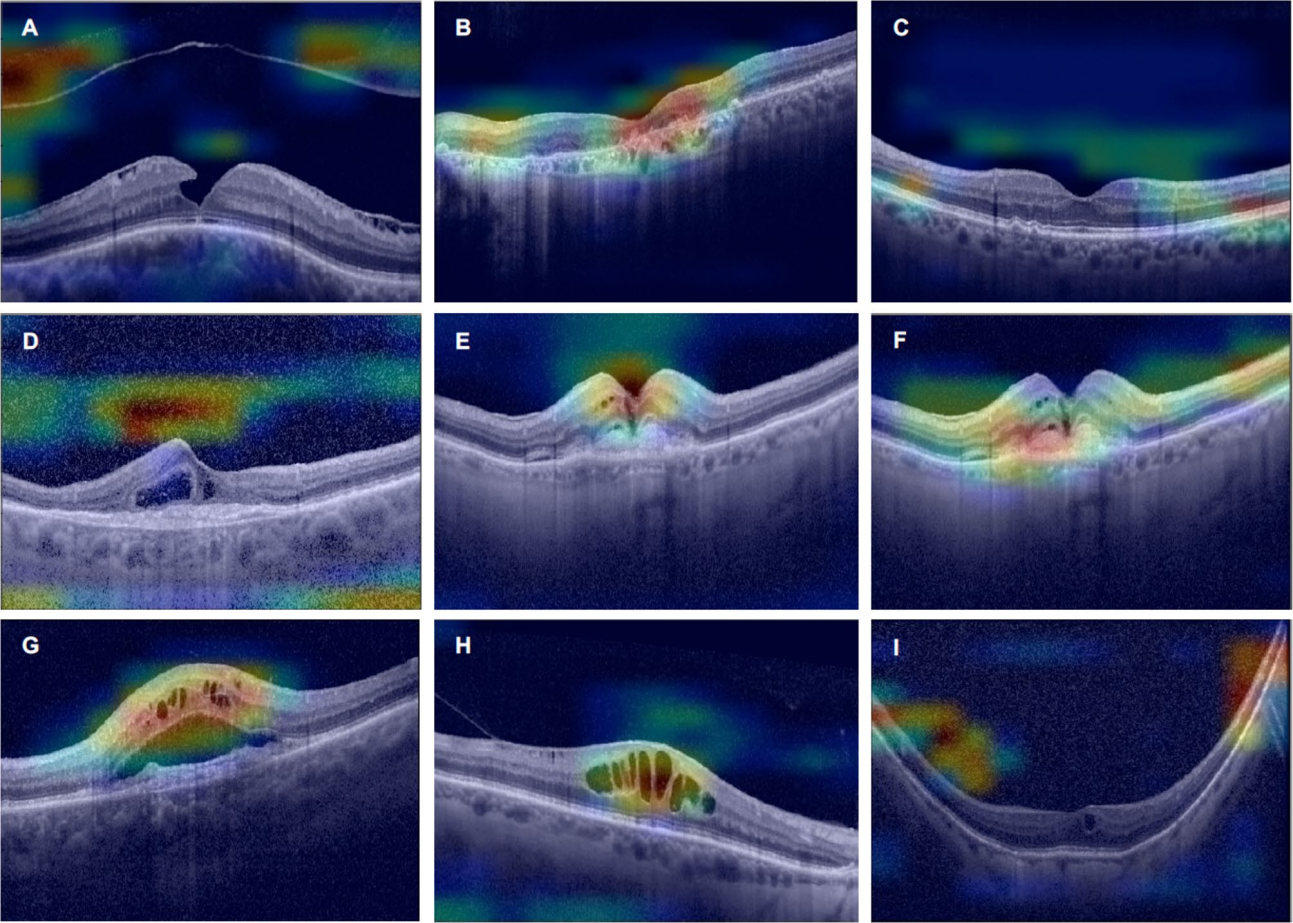
Cases in which neural networks have misclassified OCT images, providing incorrect heatmaps. (**A**) N classified as VMA. (**B**) D and BS as MNV (**C**) D classified as N. (**D**) IRF and BS classified as N. (**E**) MNV, SRF and IRF classified as MH. (**F**) SRF and VMA localized in the wrong position (**G**) IRF and SRF classified as IRF (**H**) IRF classified as MH (**I**) IRF classified as VMA.

Figure 3 shows an example of the heat maps of each pathological sign highlighting the correct localization and identification obtained by the algorithm. The system was also capable of recognizing multiple signs present in a single OCT image, as shown in the example of Grad-cam heatmaps in Fig. 5 in which the image presents three different signs. In some cases, CNNs have misclassified OCT images. This has happened only a few times as all the networks achieved very high accuracies, as highlighted before. Figure 6 shows some cases where the CNNs have produced incorrect heatmaps.

## Discussion

Nowadays OCT is an essential exam to diagnose several retinal pathologies such as DR, AMD, ERM, and MH along with other techniques, such as fundus photography and fluorescein angiography^33–37^.

Several authors developed DL systems to detect DR and diabetic macular oedema by using OCT^6,38–40^. Ting successfully trained a DL system to recognize DR, achieving remarkable results with an AUC of 0.958, a sensitivity of 100%, and a specificity of 91.1%^6^. In 2017, Lee and coworkers developed a DL system for the automated segmentation of macular oedema and showed an excellent performance in comparison with retina experts^4^. Kermany developed a CNN capable of distinguishing normal from diabetic retinopathy on 62,489 OCT images, with an impressive accuracy of 98.2%, a sensitivity of 96.8%, and a specificity of 99.6%^41^. In a study published in 2017, Schlegl and coworkers developed a fully automated method to detect and quantify intraretinal cystoid fluid (IRC) with an AUC of 0.94^40^. Abràmoff et al. created a CNN capable of recognizing DR on OCT images with an AUC of 0.98, a sensitivity of 96.8% and a specificity of 87.0%^8^.

An AI system proposed by Burlina and coworkers was capable of detecting AMD from OCT images with a good performance and an accuracy between nearly 92% and 95% in different groups^42^. Similarly, Ting et al. developed a DL system that recognizes AMD in a multiethnic population with diabetes with an AUC of 0.93, a sensitivity of 93.2% and a specificity of 88.7%^6^. Moreover, Kermany and coworkers obtained an accuracy of 96.6%, a sensitivity of 97.8% and a specificity of 97.4% to diagnose AMD from OCT images^41^. CNNs were also trained to recognize specific biomarkers for the prediction and progression of AMD disease^43–49^. Despite the availability of a large number of studies, their applicability in clinical practice is limited when considering real-world hospital conditions. Yanagihara et al.^22^ showed that one of the challenges among the others is the limited interpretability of a DL model and the non-standardized datasets, which imposes that each hospital creates its dataset.

All the studies mentioned above utilized binary classification methods to distinguish between pathological and normal OCT images and made a black-box diagnosis based on a single OCT image per patient. However, clinical diagnoses rely on identifying abnormalities across a series of OCT images taken from the same patient, as a single image may not capture all the necessary information. One possible way to address this issue is to focus on classifying signs of retinal abnormality rather than the pathologies themselves. Not many studies reported the recognition of signs. Son et al. created a system that accurately detects 15 abnormal retinal findings and diagnoses 8 major eye diseases using macula-centered fundus images. They introduced the concept of counterfactual attribution ratio (CAR) to illuminate the system's diagnostic reasoning, showing how each abnormal finding contributes to its prediction. CAR allows for quantitative and qualitative interpretation, interactive adjustments, and confirms the model's ability to identify findings and diseases similar to ophthalmologists^50^. Lu et al. proposed a DL system capable to discriminate normal images, cystoid macular oedema, serous macular detachment, ERM, and MH with an accuracy of 97%, 84%, 94%, 96% and 98%, respectively^51^. Rajagopalan et al. classified choroidal neovascularization (CNV), drusen and diabetic macular oedema (DME) with an accuracy of 97%, a sensitivity of 93%, and a specificity of 98%^52^. In another study, Kurmann implemented a machine learning method capable of recognizing various conditions in OCT B-scan images, including subretinal fluid (SRF), intraretinal fluid (IRF), intraretinal cysts (IRC), hyperreflective foci (HF), drusen, reticular pseudodrusen (RPD), epiretinal membrane (ERM), geographic atrophy (GA), outer retinal atrophy (ORA), and fibrovascular pigment epithelial detachment (FPED). They developed the DL system using 23,030 OCT B-scan images, achieving remarkable results^53^.

Our DL models like those of Kurmann were trained on small datasets (which could be more easily acquired within a single hospital) and were designed to detect a variety of retinal abnormalities in multiple input images from the same patient. By identifying individual abnormality signs, they replicate the deductive process followed by the ophthalmologist in diagnosing ocular pathologies, rather than solely relying on the results generated by a black-box DL learning model. The clinical procedure, where doctors have access to multiple images and use them to assess the presence of all nine signs, was aimed to be replicated. A comprehensive understanding of the process of sign identification was sought by observing the classifier output, including class probabilities, and analyzing the heatmaps. This method also minimizes the total number of images typically required to distinguish between different pathologies. Whereas the creation of a CNN model specific to a particular pathology requires many images associated with that pathology, the identification of a sign could be accomplished by using images that are common to different pathologies. Therefore, our approach drastically reduced the overall time necessary for image collection. The VGG-16 is based on a relatively simple CNN design consisting of a series of stacked convolutional layers followed by max pooling and then fully connected layers at the end. This simple architecture means that VGG-16 has a smaller number of parameters compared to ResNet and Inception, which have more complex architectures with skip connections, residual blocks, and inception modules that enable them to learn more complex features.

Lee et al. demonstrated that CNN can be successfully used to distinguish normal OCT images from patients with AMD^20^. The authors extracted 2.6 million OCT images from normal subjects and AMD patients. Of these, 80,839 images were selected to train a CNN model, while 20,163 images were used to validate it. The architecture chosen was a modified version of the VGG-16 network. ROC curves were created at the image level, macular level and patient level, and the AUCs achieved were 92.78%, 93.83%, and 97.45%, respectively. Choi et al. trained and validated three CNNs to classify normal, high myopia, and other retinal disease groups based on OCT images^21^. The authors adopted three specific architectures (VGG-16, ResNet-50, and Inception-v3) as a backbone and developed models to perform image classification. The best AUCs of the three CNNS models were 99.9% for VGG-16, 100.0% for ResNet-50 and 96.1% for Inception-v3.

Despite using simpler architecture, comparably to the previous works, our models achieved a high level of accuracy on both the training and test sets, ranging from 93 to 99%, for identifying healthy retinas and eight specific pathological signs. The similar model performance on both the validation and the test sets, suggests that our nine models were robust, did not overfit during the training and learnt to capture the underlying patterns related to retinal abnormality signs so that they could classify well also unseen data. Finally, the relatively high performance of our models, demonstrated by the results, underlines the potential capacity of these models to identify single or multiple signs in OCT images.

We acknowledge that VGG16 is not the most recent architecture and might not have the highest accuracy, and that some approaches might achieve some more, however, even such achieving the clinically relevant results for retinal deterioration sign detections. We believe also that utilizing VGG's general-purpose nature and the abundance of tutorials and implementations available is its advantage. Apart from that, the time it takes for the models to classify the image depends mostly on the characteristics of the computer used. In our settings (with our computer), the time to classify the uploaded image in the system was 2.2 s. The real-time classifier performance holds significance, it is not the primary factor determining its clinical relevance. While achieving extremely high accuracy (e.g., close to 100%) may be unrealistic or impractical for some medical imaging tasks, it is important to focus on achieving clinically relevant accuracy levels^54,55^.

These levels may vary depending on the specific medical task, the potential impact on patient care, and the specific use-case scenario^56^.

Markedly, our approach could allow ophthalmologists to analyze each OCT image separately, as not all signs might be discernible in every image. Furthermore, since the system could identify individual signs rather than being restricted to single retinal pathologies, it could serve as a diagnostic aid for a much wider range of pathologies presenting a different combination of these signs. On the other hand, the classification of singular signs might be considered a drawback, as it still requires the intervention of the ophthalmologist to identify a pathology as required in automated screening applications.

## Conclusions

The development of DL models that can accurately and automatically detect abnormal retinal signs from OCT images has significant implications for patient care. Although many studies have focused on the classification of ocular pathologies, our study aimed to identify individual signs related to a pathology, which allows the ophthalmologist more room to provide additional interpretation to reach a correct diagnosis. Our system achieved high accuracy in identifying healthy retinas as well as specific pathological signs making it a useful diagnostic aid for a wide range of pathologies. The Grad-Cam visualization enhanced the interpretability of our CNN's results, allowing ophthalmologists to assess the model's efficacy. While the need for a considerable amount of labelled OCT images to train the model remains a challenge, our approach reduced the time required to create separate datasets for each retinal pathology. In our study, we utilized the VGG16 architecture. Despite its accessibility, there are potential drawbacks associated with it. Exploring the feasibility of employing newer deep learning architectures and comparing their performance could enhance the integration of machine learning into the diagnostic process. Overall, our study demonstrated the potential of DL models in improving the diagnosis of ocular pathologies and supporting clinical decision-making.

## Data Availability

The datasets generated and analyzed during the study are not publicly available due to privacy constraints. The data may however be available from the University of Trieste subject to local and national ethical approvals. In addition, we have made the models and relevant code available upon request. Any requests should be sent to the corresponding author.
